# Bread Composition and Dietary Fibre Intake: Modelling Consumption Patterns and Substitution of White with Wholegrain Bread

**DOI:** 10.3390/nu17223523

**Published:** 2025-11-11

**Authors:** Hristo Hristov, Živa Lavriša, Igor Pravst

**Affiliations:** 1 Nutrition and Public Health Research Group, Institute of Nutrition, Koprska Ulica 98, SI-1000 Ljubljana, Slovenia; ziva.lavrisa@nutris.org; 2 National Institute of Public Health, Trubarjeva Cesta 2, SI-1000 Ljubljana, Slovenia; 3 Biotechnical Faculty, University of Ljubljana, Jamnikarjeva Ulica 101, SI-1000 Ljubljana, Slovenia; 4 VIST—Faculty of Applied Sciences, Gerbičeva Cesta 51A, SI-1000 Ljubljana, Slovenia

**Keywords:** bread, dietary fibre, wholegrain, dietary intake modelling

## Abstract

Background/Objectives: Inadequate dietary fibre intake remains a public health concern across Europe, particularly for adolescents. Bread is a widely consumed staple and a potential vehicle for improving dietary fibre intake. This study aimed to quantify the dietary fibre content of commonly consumed breads and assess their contribution to daily dietary fibre intake in the Slovenian population, with a focus on adolescents. Methods: A total of 58 bread samples were analysed using an accredited enzymatic-gravimetric method. Consumption data were drawn from the nationally representative SI.Menu dietary survey. Results: A substantial variation in dietary fibre content was observed across bread types, with wholegrain breads providing the highest levels (mean: 7.5 g/100 g) and white wheat breads the lowest (2.9 g/100 g). In adolescents, white wheat bread was most often consumed, contributing disproportionately to total bread intake and limiting dietary fibre intake. We modelled two substitution scenarios, replacing 30% and 50% of white wheat bread with wholegrain bread, which resulted in mean dietary fibre increases of 1.4 g/day and 2.0 g/day, respectively. Conclusions: These findings demonstrate that realistic bread substitution strategies—particularly in institutional settings such as schools—could significantly improve dietary fibre intake in youth populations. The study also underscores the need for clearer regulatory definitions and labelling of wholegrain bread, especially in non-prepacked products.

## 1. Introduction

The rising prevalence of non-communicable chronic diseases, including cardiovascular disease, type 2 diabetes, and colorectal cancer, has increasingly drawn attention to the role of dietary fibre in disease prevention and overall public health [1,2,3]. Despite extensive evidence highlighting the benefits of fibre-rich diets, such as improved glycaemic control, reduced serum cholesterol, enhanced gastrointestinal health, and potential protective effects against certain cancers [2,4], dietary fibre intake remains suboptimal in many European populations, including Slovenia [3,5,6].

Bread, as an abundantly consumed staple food, represents a critical vehicle for dietary fibre intake. In the United Kingdom, for instance, bread contributes to approximately 17–21% of daily dietary fibre intake across all age groups, underscoring its importance as a dietary fibre source [5]. In Slovenia, bread and grain products have also been identified as the main dietary fibre contributors [6,7,8]. These findings are based on data from the nationally representative Slovenian National Food Consumption Survey, SI.Menu 2017/2018, which was conducted between March 2017 and April 2018 following the EFSA Guidance on EU Menu Methodology. The cross-sectional survey included Slovenian residents aged 10–74 years and applied a two-stage probability sampling design stratified by region and degree of urbanisation to ensure population representativeness. Dietary data were collected across all 12 statistical regions, enabling robust estimation of food and nutrient intake patterns, including the contribution of breads and cereal products to total dietary fibre intake in the Slovenian population.

However, shifts in dietary habits, increased consumption of refined bakery products, and limited consumer awareness of bread’s dietary fibre content have highlighted the need to better understand the actual contribution of particular bread types to daily dietary fibre intake. Given bread’s widespread consumption and nutritional relevance as a major source of dietary fibre, it represents a practical target for improving population-level dietary fibre intake through food-based interventions. This is particularly pertinent in Slovenia among adolescents, whose dietary fibre intake often falls below recommended levels and who consume a large share of their daily diet within structured school meal settings [6].

Systematic monitoring and analytical determination of the dietary fibre content in popular breads currently available on the market are essential for capturing the impact of reformulation efforts and evolving market trends on population-level dietary fibre intake [9], which is also very relevant for Slovenia [6]. Such data are vital for more accurate estimation of bread’s contribution to dietary fibre intake and for planning public health interventions, and also for supporting the integration of reliable, up-to-date values into national food composition databases, which are typically based on generic food profiles [10].

Improving the nutritional composition of bread and encouraging wholegrain bread consumption involve a combination of activities, including food reformulation, consumer education, and strategic labelling. The UK’s government-led initiatives, such as the salt reduction programme and the consideration of mandatory folic acid fortification, have contributed to the improved nutritional quality of bread [5]. Additionally, front-of-pack labelling systems have been shown to effectively guide consumers towards healthier bread choices. Countries like Denmark and the Netherlands have successfully promoted wholegrain bread consumption through coordinated national campaigns and supportive labelling initiatives [11,12,13]. While the national Rules on the Quality of Bakery Products define bread types in Slovenia [14], and reformulation initiatives related to decreasing the salt content in bread have been established [15], the labelling of the dietary fibre content of prepacked-foods is voluntary across the whole European Union. Additional challenges arise with non-prepacked breads, which make up most of the bread sold in Slovenia. These products lack ingredient lists and nutritional declarations, leaving consumers without essential information. While national regulations require at least 80% wholegrain flour in breads labelled as ‘wholegrain’, there is no standard for minimum dietary fibre content. This regulatory gap complicates dietary fibre content assessment for consumers, researchers, and food control authorities alike, and may hinder informed decision-making, trust, and efforts to promote healthier food choices.

Therefore, the aim of this study was to provide a comprehensive analytical assessment of dietary fibre content in commercially available breads in Slovenia; to evaluate their contribution to population-level dietary fibre intake; and to estimate the potential benefits of substituting white wheat bread with wholegrain bread in daily consumption patterns. Substitution modelling focused on adolescents, a population group identified in previous national studies as having the highest prevalence of inadequate dietary fibre intake and relying on bread as a key source of dietary fibre. The findings of this study are intended to support evidence-based public health policies, inform product reformulation by the food industry, and promote dietary fibre-rich bread consumption.

## 2. Materials and Methods

### 2.1. Bread Sample Collection

The sampling for this study was taken from a larger, nationally representative sample of breads available in Slovenia (*n* = 178), which were originally collected to assess sodium levels in bread available on the market; the detailed sample collection has been previously described [16]. In brief, a stratified sampling approach was based on the household consumption categories provided by the Statistical Office of the Republic of Slovenia [17]. Bread categories with higher reported household consumption were prioritised to ensure that the most consumed types were adequately represented. Purposive sampling was employed to capture a broad representation of the bread types available on the market, including suppliers from different statistical regions and retail formats. Sampling was taken in (a) 5 large food retailers; and (b) 32 smaller bakeries across Slovenia. In the large retail stores, all the available bread types were sampled. In contrast, the sampling in small bakeries was limited to non-prepacked breads sold as ‘white bread’ or ‘wholegrain bread’, with a focus on products with the highest sales turnover. Purchases were made discreetly, with the researcher posing as a regular customer and requesting the most popular white or wholegrain bread.

A total of 58 bread samples were selected for dietary fibre content analysis. To reflect their dietary relevance and expected variability, sampling focused on commonly consumed bread types: white wheat, half-white wheat, dark wheat, wholegrain, and mixed breads. Mixed and wholegrain breads, known for their greater variability in dietary fibre content, were intentionally sampled in larger numbers to improve estimate precision and capture the range of compositional differences within these categories [18]. Of the 32 small bakeries visited, only three reported that they do not offer wholegrain bread. Consequently, wholegrain bread samples were purchased from the remaining 29 bakeries—one sample from each. All 29 samples marketed as wholegrain bread by small bakeries were subsequently reassessed and recategorized, using the methodology described in Section 2.2, to accurately reflect the wholegrain bread category, as the technological and regulatory standards do not always align with market practices or sales intentions.

### 2.2. Bread Type Classification

The bread samples were classified according to the Slovenian Rules on the Quality of Bakery Products [14]. Under these regulations, mixed wheat bread must contain at least 51% wheat flour; rye wholegrain bread, at least 80% rye flour; and wholegrain wheat bread, a minimum of 80% wholegrain wheat flour. White, half-white, and dark wheat breads are differentiated based on the mineral (ash) content of the wheat flour used [19]. While labelled packaging and regulated product names enabled reliable classification of the bread types in large retailers, this was more challenging for the non-prepacked breads sold in small bakeries. Visual inspection of products marketed as ‘wholegrain bread’ in these bakeries suggested that many likely did not meet the required 80% wholegrain flour threshold. In such cases, the bread type classification was based on the product name listed on the official invoice, the visual characteristics (e.g., the presence of seeds), and the sample’s composition. To support its classification, we also estimated a minimum expected dietary fibre content for wholegrain bread. This threshold was estimated by modelling the dietary fibre content, based on three key assumptions: (a) the regulatory minimum requirement of 80% wholegrain wheat flour in wholegrain bread, with the remaining 20% modelled as white wheat flour; (b) the minimum dietary fibre content of both wholegrain and white wheat flours, as derived from the national branded food composition database (CLAS—Composition and Labelling Information System) [20], and (c) the maximum practical flour:water dissolution (1:0.7) [21]. Considering these conservative estimates, breads containing less than 5.3 g of dietary fibre per 100 g are unlikely to contain the required 80% wholegrain flour, and therefore do not meet the national criteria for being labelled as “wholegrain” [19].

Following this methodology, the majority of samples offered as ‘wholegrain’ in small bakeries were re-categorised into other bread types, mostly mixed breads (with or without seeds) (Supplementary Table S1).

### 2.3. Sample Preparation and Laboratory Analyses

After purchase, the fresh bread samples were hermetically vacuum-sealed in moisture-barrier polyethylene foil and transported to the external laboratory, Mérieux NutriSciences (Resana, Italy). The quantification of total dietary fibre (TDF) was conducted using the accredited official method [22] (Accreditation No. MP 2135 rev 6 2021), a procedure also utilised by food control authorities to verify the accuracy of nutrition labelling. In brief, this is the analytical enzymatic–gravimetric method, which includes enzymatic digestion of the sample, followed by ethanol precipitation. Method involves three enzymes, supplied by Megazyme Ltd. (Bray, Ireland): heat stable alpha-amylase (supplier code: E-BLAAM), Protease (supplier code: E-BSPRT), and Amyloglucosidase (supplier code: E-AMGDF). Residues were filtered, dried, incinerated, and weighed. Measured fibre content includes both soluble and insoluble dietary fibre and was calculated based on the difference between filtered residue and ash/protein mass and is expressed as g/100 g. The method’s quantification limit for dietary fibre in bread and similar matrices was 0.5 g per 100 g.

### 2.4. Assessment of Dietary Fibre Intakes

The dietary fibre intake from the bread was assessed using consumption data from the most recent national cross-sectional dietary survey, SI.Menu, conducted in accordance with the EFSA’s Guidance on European Union Menu Methodology [23]. The study details about the methodology and sample characteristics have been published elsewhere [24]. In short, the participants were stratified into three age groups: adolescents (10–17 years old), adults (18–64 years old), and older adults (65–74 years old). A total of 2280 subjects were selected using the Central Register of Population (CRP) of Slovenia according to age, sex, and place of residency. The selected residents were visited by researchers, who checked the eligibility of the respondents and collected the required information, including the food propensity questionnaire and two 24 h dietary recalls. The survey was completed by a total of 59% of the invited participants (*n* = 1345). The study protocol was approved by the National Medical Ethics Committee of the Republic of Slovenia (KME Approval No. 0120-337/2016, issued on 19 July 2016).

Bread consumption data was extracted from the 24 h dietary recalls in the SI.Menu study dataset. The reported foods were assigned to the appropriate bread type and linked to the food composition data established in this study. Individual intakes of dietary fibre from bread were calculated by multiplying the reported amount of bread consumed per eating occasion by the median dietary fibre content analytically determined for the respective bread type. In total, 3045 bread consumption entries were recorded in the SI.Menu dataset (across both 24 h recalls) for all 1345 participants, most commonly (*n* = 1140) for white wheat breads.

### 2.5. Modelling of Hypothetical Substitution of White Wheat to Wholegrain Bread Scenarios

We modelled two hypothetical dietary intervention scenarios involving the substitution of white wheat bread with wholegrain bread in adolescents. Scenario A replaced 30%, and Scenario B replaced 50% of white wheat bread intake with a wholegrain alternative, without altering the consumption of other bread types. Daily bread intake was recalculated accordingly, and the contribution of bread to total dietary fibre was estimated using the described methodology. Within-subject differences were calculated by subtracting baseline dietary fibre intake (no substitution) from intake under each substitution scenario, generating individual-level change variables. The mean of these differences across the participants represents the average expected increase in daily dietary fibre intake for each scenario.

### 2.6. Data Processing and Statistical Analysis

For statistical analyses and data representation, IBM SPSS Version 27 (IBM SPSS, IBM Corp., Armonk, NY, USA) and STATA (Version 17.0; StataCorp LLC, College Station, TX, USA) were used. The Multiple Source Method (MSM) V1.0.1 [25]—a statistical program developed by the Department of Epidemiology of the German Institute of Human Nutrition, Potsdam-Rehbrücke, Germany, was used to estimate the habitual intake distribution of the amount of bread and the respective bread categories, and of dietary fibre and energy intakes. Day-to-day inter- and intra-individual variations in the habitual intake distribution were modelled using the Multiple Source Method (MSM), where age, sex and bread frequency intakes on daily basis were used as covariates.

Descriptive data are presented as means and standard deviations for continuous variables, and as counts and percentages for categorical variables. For all breads, and by bread category, median, minimum, and maximum values were also reported, with dietary fibre content expressed in grams per 100 g of bread. Habitual intake data (the amount of all bread and by category, as well as dietary fibre and energy intakes) were calculated separately for males and females and across age groups, and are presented as means per day with standard deviations (SD). Dietary fibre intake was calculated in grams per day, and in grams per 100 g of consumed bread per day. Additionally, we calculated and report the number of non-reporters of bread intake by age group and sex.

To identify the potential determinants (sex, age group, BMI, degree of urbanisation, geographic region, International Physical Activity Questionnaire (IPAQ) score, household income, education, smoking status) explaining the variation in daily bread consumption, we applied the Least Absolute Shrinkage and Selection Operator (LASSO) regression method, as recommended by Freese et al. (2016) [26]. This procedure is considered one of the most appropriate for variable selection in linear mixed-effects models. Although based on linear regression, LASSO introduces shrinkage by minimising an objective function that penalises some coefficients towards zero, effectively addressing multicollinearity and supporting variable selection. The final model included a small subset of predictors—those with nonzero coefficient estimates influencing the response variable. The most suitable LASSO model was selected using the adaptive method. This approach allows for identifying the most influential sociodemographic and lifestyle predictors of bread consumption while reducing model complexity and overfitting, thereby improving interpretability of the results.

## 3. Results

### 3.1. Dietary Fibre Content of Different Bread Types

Dietary fibre content was analytically determined in 58 breads, of which 27 were collected from retail shops, and 31 from small bakeries. Of the 29 breads originally purchased as wholegrain from small bakeries, only 3 met our criteria for wholegrain bread (see methodology Section 2.2). The remaining breads were reclassified to other bread types, as reported in Table S1.

We observed substantial variation in dietary fibre content across the different bread types (Table 1 and Table S2). White wheat bread showed the lowest dietary fibre content, with a mean of 2.9 g/100 g. In contrast, wholegrain bread had the highest dietary fibre content, with a mean of 7.5 g/100 g, and a broad range from 5.3 to 9.6 g/100 g. Half-white and dark wheat breads exhibited intermediate mean dietary fibre levels, of 3.7 g and 4.4 g/100 g, respectively. Notably, dietary fibre variability increased slightly with darker breads, as indicated by a standard deviation of 0.8 g for dark wheat bread. Mixed breads had a relatively high average dietary fibre content of 4.6 g/100 g. To provide more insights, we also separately assessed mixed breads with and without seeds. The seed breads had a higher mean dietary fibre content and variability (4.8 g/100 g, SD 1.5 g) than those without seeds (4.4 g/100 g, SD 0.6 g). The dietary fibre content range was widest in the mixed bread category (3.3–8.3 g/100 g), suggesting high heterogeneity due to ingredient diversity. More detailed results with regard to the purchase locations are presented in Table S2.

### 3.2. Bread Consumption and Contribution to Dietary Fibre Intake

Table 2 presents the contribution of different bread types to the estimated habitual mean total bread intake across adolescents, adults, and older adults in the Slovenian population, disaggregated by sex. White wheat bread ranked as the primary contributor to daily bread intake across most of the investigated population groups. In adolescents, white wheat bread accounted for 54.6% of the daily bread intake of males, and 52.1% of the intake of females. In adults, the contributions were 39.4% for males and 34.6% for females, while in older adults the values were 24.9% and 25.4%, respectively. The contribution of dark wheat bread was notably higher in older adults (39.8% in males and 30.5% in females) than in adolescents (18.3% and 15.6%, respectively). Wholegrain bread had even lower contribution to bread intake—from about 10% in adolescents (10.6% in males and 7.9% in females), to about 15% in older adults (13.4% in males and 17.5% in females). Non-reporting of white wheat bread intake over two 24 h recalls was highest among older adult females (67.5%), followed by older adult males (64.6%) and adult females (63.8%). In contrast, adolescent males had the lowest non-reporting rate (37.5%), indicating more frequent consumption in this group. The proportion of true non-consumers (those reporting no intake of any bread type) was very low (below 4.3%) across all age groups, underscoring bread’s role as a staple food widely consumed in the population.

Table 3 summarises the findings concerning the mean dietary fibre content in consumed bread across various sociodemographic and lifestyle variables. The Least Absolute Shrinkage and Selection Operator (LASSO) method was applied to identify the significant determinants for the dietary fibre content in consumed bread. Sex was found to be an important predictor of bread dietary fibre content; females showed higher values (3.72 g/100 g) than males (3.50 g/100 g). Regional differences were also identified as important, with individuals in the eastern region consuming more dietary fibre rich bread (3.62 g/100 g day) than those in the western region (3.58 g/100 g day). The degree of urbanisation was also associated with the dietary fibre content in consumed bread, with those in intermediate areas showing the highest content (3.73 g/100 g). Education level showed a positive association, with those having a higher level of education consuming bread with more dietary fibre (4.10 g/100 g) than those with a lower level (3.61 g/100 g day). Employment status was also a relevant predictor, with unemployed individuals exhibiting the lowest bread dietary fibre content (3.59 g/100 g).

Low physical activity, as measured by the International Physical Activity Questionnaire (IPAQ) score, was associated with slightly lower dietary fibre intake. These results suggest that variables such as sex, region, urbanisation, education, employment, and physical activity are associated with dietary fibre intake from bread.

Table 4 presents the contribution of different bread types to the estimated habitual mean total dietary fibre intake (TDF) from bread in Slovenian adolescents, adults and older adults, stratified by sex. Bread-related TDF intake increased with age; we observed higher values in older adults (4.8 g/day for males and 4.4 g/day for females) and adults (5.0 g and 4.0 g, respectively), compared to adolescents (4.7 g and 3.2 g, respectively). White wheat bread was the major contributor to TDF in adolescents, accounting for 46.0% of bread dietary fibre intake in males, and 41.0% in females. However, the relative contribution of white wheat bread declined with age: in older adults it accounted for only 24.2% of bread dietary fibre intake in males, and 20.6% in females. In contrast, the contribution of wholegrain bread increased with age and became the primary source of TDF in adults and older adults. In older adults, wholegrain bread contributed to 36.3% of bread dietary fibre intake in males and 39.7% in females—the highest contribution of all bread types in these groups. Adolescent males consumed notably more dietary fibre from wholegrain bread (1.2 g/day) than females (0.6 g/day). In adults and older adults, the absolute intake of dietary fibre from wholegrain bread was similar between the sexes, but its relative contribution was higher in females. Wholegrain bread was the top TDF contributor in adult and older adult females, (36.2% and 39.7%, respectively). These patterns highlight a clear shift in bread type consumption influencing dietary fibre intake across the lifespan. Adolescents rely more heavily on white wheat bread, whereas adults, particularly older adults, obtain a greater proportion of bread as wholegrain option. This shift may reflect age-related changes in dietary preferences or intentional dietary choices, such as increased wholegrain consumption for health-related reasons.

### 3.3. Estimating the Dietary Fibre Intake Impact of Partial White-to-Wholegrain Bread Substitution

**Figure 1:**
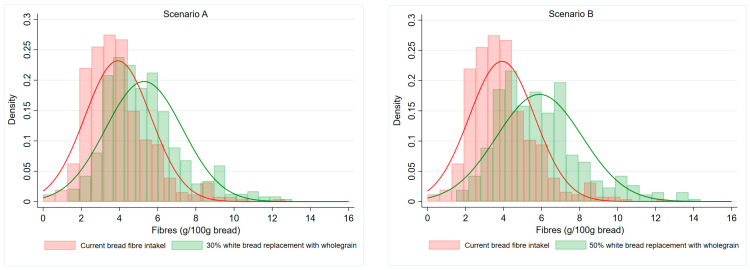
Histograms of dietary fibre intake from bread in the baseline diet (red bars) compared with modelled substitution scenarios: 30% replacement (**left**, Scenario A) and 50% replacement (**right**, Scenario B) of white wheat bread with wholegrain bread (green bars).

Using consumption data from adolescents, we modelled two realistic scenarios: replacing either 30% (Scenario A) or 50% (Scenario B) of consumed white wheat bread with a wholegrain alternative, without altering the intake of other bread types. The modelling was applied to the entire adolescent sample, including both males and females. While the baseline mean contribution of bread to the habitual daily dietary fibre intake (actual diet) was 3.92 g (SD 1.72 g), Scenario A (30% replacement) resulted in 5.29 g (SD 2.02 g), and an even higher intake was estimated in Scenario B (50% replacement): 5.89 g (SD 2.25 g). The increase to the daily dietary fibre intake was 1.37 g (SD 0.92 g) in Scenario A, and 1.97 g (SD 1.20 g) in Scenario B. The results of the populational distribution of daily bread dietary fibre intakes are presented in Figure 1. In both scenarios, the distribution of dietary fibre intake shifts to the right (green bars) compared to the actual diet (red bars), indicating an increase in dietary fibre intake. The green bars reflect the modelled dietary fibre intake from bread under the substitution scenarios, showing a noticeably higher and broader distribution, particularly in the 50% replacement scenario (Scenario B). The peak of the distribution flattens and moves toward higher dietary fibre values, suggesting a more even spread of intake and greater overall dietary fibre consumption in adolescents. These projections indicate that even partial replacement of white wheat bread with wholegrain bread could substantially improve dietary fibre intake from bread in this population.

## 4. Discussion

This study provides a comprehensive national analysis of dietary fibre content in commercially available Slovenian breads and evaluates bread’s contribution to overall dietary fibre intake, representing the most detailed published assessment of this topic in Slovenia. Our findings reveal substantial variability in dietary fibre content across bread types, as well as inconsistencies in the marketing and classification of non-prepacked breads.

Mixed-flour breads exhibited the highest heterogeneity in dietary fibre content (3.3 to 8.3 g/100 g bread), which can be attributed to the wide range of flour types used in their formulation, each contributing differently to the overall dietary fibre content. The addition of other ingredients also considerably affects the nutritional composition. Seeds can be added to boost the dietary fibre content of otherwise refined mixed-flour breads, or to help position products perceived as ‘wholegrain’ to appeal to health-conscious consumers [27]. The notable differences in dietary fibre content between breads from large retail stores and small bakeries, particularly in mixed flour and seeded varieties, reflect variations in the product formulation, quality control, and interpretation of what constitutes wholegrain bread. As expected, wholegrain breads from large retailers contained high levels of dietary fibre (mean: 7.6 g/100 g; SD: 1.5 g), due to the national regulatory requirement mandating at least 80% wholegrain flour in such products. This is further supported by business-to-business competition and the probable greater likelihood of regulatory oversight in large retail settings. The observed mean dietary fibre content of wholegrain breads also corresponds to the American Heart Association’s recommendation of an optimal dietary carbohydrate-to-fibre ratio of ≤10:1 [28]. In contrast, wholegrain breads offered in small bakeries contained significantly lower levels of dietary fibre (mean: 4.6 g/100 g; SD: 1.2 g), often falling below the minimum theoretical dietary fibre content expected for breads made with at least 80% wholegrain wheat flour. Our findings suggest that staff in these bakeries may offer darker-coloured breads or those containing seeds when customers request a wholegrain option, regardless of the actual flour composition. We estimated that only about 10% of breads labelled or offered as wholegrain in small bakeries were truly wholegrain (Table S1). These findings underscore the need for better staff education, more rigorous food authority controls, and clearer regulatory guidance on the labelling of non-prepacked breads.

Variability in dietary fibre content and labelling practices reflects broader challenges in the bread market, as highlighted in a Quebec study. The research found that most best-selling breads, including those labelled as ‘multigrain’, contained less than 2 g dietary fibre per slice, below the thresholds for dietary fibre claims. Furthermore, only 73% of multigrain breads labelled ‘100% whole grain’ qualified as a ‘source of dietary fibre’ and just 38% met the criteria for being ‘high in dietary fibre’ [29]. Countries, including Slovenia, may benefit from supporting more transparent communication of wholegrain content, particularly for non-prepacked breads. While current practices are not necessarily misleading, producers may emphasise certain aspects, such as the presence of seeds or grains, over the actual dietary fibre content or ‘wholegrain’. Introducing clearer criteria, such as minimum dietary fibre thresholds for wholegrain breads, could help ensure more consistent messaging across different sales channels. This would support consumer trust and promote dietary choices in line with public health recommendations on dietary fibre intake.

Our study is also in agreement with previous research [9], demonstrated that standardised sampling and laboratory analysis can generate valuable, up-to-date data on the nutritional composition of foods available on the market. This is particularly important for assessing dietary fibre intake from bread, where generic food profiles may not reflect the diversity of commonly consumed products. Incorporating current, locally derived analytical data into national food composition databases can improve the accuracy of dietary assessments and better inform nutrition policies and consumer guidance. As noted by Pennington (2008) [10] and Lee et al. (2008) [9], reliance on outdated or non-local food composition data introduces a risk of error which can compromise public health strategies. Our findings underscore the need to routinely generate and integrate such data to ensure dietary policies are relevant, evidence-based, and aligned with actual consumption patterns. This is particularly the case for staple food categories, where composition is particularly affected by innovations, reformulation efforts, and the changing expectations of consumers.

Promoting wholegrain consumption has become a key public health priority in many European countries, due to its well-established health benefits. The Danish Whole Grain Partnership [30] is a successful public–private initiative which has significantly increased wholegrain consumption in Denmark through coordinated efforts in product reformulation, consumer education, and clear labelling, which has been followed by other countries [11]. Building on this model, Slovenia joined the EU-funded WholEUGrain project in 2019, to adapt and implement similar strategies aimed at promoting wholegrain intake at the population level [31]. Our findings show that, with increasing age, bread consumption patterns shift from white wheat bread to mixed and wholegrain varieties. This is likely driven primarily by traditional eating habits established earlier in life, as older adults tended to consume less white bread when they were younger due to its lack of availability, favouring more traditional, less refined breads. These longstanding preferences, likely shaped by environmental, cultural and generational factors, appear to play a potentially more significant role than health motivations in influencing their current choices [32]. In contrast, younger populations such as adolescents are more accustomed to refined white bread, reflecting modern dietary trends, availability and taste preferences [33], which makes white bread the bread of choice in this population group. What is concerning is that this trend will likely to continue into their adulthood.

Adolescence is a critical period for establishing lifelong eating habits, so encouraging wholegrain bread consumption can have lasting health benefits. This population group is also particularly relevant for policy-driven dietary interventions. School-aged children in Slovenia receive meals provided through school programmes, offering a valuable opportunity to implement healthier options, such as wholegrain bread, in a controlled setting where nutrition can be better managed and monitored. This makes schools an ideal environment for nutritional education, promoting dietary improvements and positively influencing dietary fibre intake in children and adolescents [34]. With the consideration of the official national Nutrition Guidelines for Educational Institutions [35], meals provided in the school environment contribute to 35–55% of daily energy consumption. Based on these findings, we modelled a hypothetical dietary intervention in which white wheat bread served in schools was partially replaced with a wholegrain alternative. The results demonstrate that even a partial substitution could substantially improve dietary fibre intake in adolescents. Replacing 30% of white bread with wholegrain increased the mean daily dietary fibre intake by 1.4 g, while a 50% replacement resulted in a 2 g increase. Considering the total daily dietary fibre intake in this population group [6], this represents an improvement of approximately 7–10%. Such an intervention could meaningfully support the development of healthier eating patterns and the improved nutrient intake essential for long-term health. A study by Revheim et al. (2025) [36] shows that increasing wholegrain consumption in general, and reducing the intake of refined white bread, could promote weight regulation from middle to late adulthood. For these reasons, targeted activities and policy measures are needed to support and promote the substitution of white wheat bread with wholegrain alternatives as part of a comprehensive strategy to increase wholegrain consumption in young people. School meal programmes, educational initiatives, and bread reformulation incentives could be valuable tools to encourage these shifts and help to address the ongoing challenge of insufficient dietary fibre intake in this age group.

A key strength of this study is in the fact that dietary intakes were not calculated using generic food composition databases or labelling data, but are based on the analytically quantified content of dietary fibre in quite a large sample of foods. By integrating these values with individual-level consumption data from the national SI.Menu survey, the study offers a more accurate and context-specific estimate of bread’s contribution to dietary fibre intake. The focus on bread, which is a major dietary fibre source, along with the modelling of practical substitution scenarios in adolescents, adds relevance for planning future public health interventions.

Some study limitations should also be mentioned. Dietary intake modelling was based on two 24 h dietary recalls per participant, which may not have fully captured their usual intake and could lead to under- or overestimation of bread consumption and dietary fibre intake at the individual level. Additionally, while the bread samples were selected to reflect commonly consumed products across major retail chains and small bakeries, the purposive sampling approach may not fully represent all the available bread types on the Slovenian market. The absence of a legally required minimum dietary fibre content for wholegrain bread limited our ability to reliably categorise non-prepacked breads sampled in small bakeries. To address this, we established internal minimum criteria for dietary fibre content, based on the regulatory requirement of at least 80% wholegrain wheat flour in such products, the known dietary fibre content of wholegrain and white wheat flour, and the maximum practical dilution of flour with water. While the threshold applied in our classification aligns with existing international criteria, and is supported from both technological [37] and public health perspectives [28], it is likely that we overestimated the proportion of wholegrain breads in comparison with the regulatory required 80% wholegrain flour alone. We should also mention that we did not assess the data on bread production methods or specific ingredient compositions because this is not available for non-prepacked foods. Moreover, the analytical results were limited to total dietary fibre content, without distinguishing between soluble and insoluble fractions. This may be particularly relevant for seeded and multigrain breads, where dietary fibre composition is more heterogeneous and may influence nutritional quality and functional properties.

## 5. Conclusions

In conclusion, this study highlights the substantial variability in the dietary fibre content of different bread types on the Slovenian market, particularly within mixed breads. We also observed a critical issue for consumers seeking wholegrain breads in small bakeries: they are often offered mixed-flour breads with significantly lower dietary fibre content. This finding underscores the need for better staff education, stricter control by food authorities, and clearer regulatory definitions for wholegrain bread, particularly in the non-prepacked segment. In addition, this study provides valuable data on dietary fibre intake from bread, supporting future policy and public health initiatives. By combining analytically determined dietary fibre content with national dietary intake data, we provided a more accurate estimate of bread’s contribution to population-level dietary fibre intake. The study results suggest that even partial substitution of white bread with wholegrain varieties could meaningfully enhance daily dietary fibre intake in adolescents. Given that a substantial share of adolescents’ food intake occurs in the school setting, introducing wholegrain bread through school meals represents a particularly relevant intervention strategy. The feasibility and implementation of such an intervention should be further explored.

## Figures and Tables

**Table 1:** Descriptive statistics of dietary fibre content in sampled breads by bread type.

Bread Categories	*n* (%)	Mean (SD) (g/100 g)	Median	Min	Max
White wheat bread	7 (12.1)	2.9 (0.4)	3.0	2.4	3.5
Half-white wheat bread	6 (10.3)	3.7 (0.4)	3.7	3.2	4.2
Dark wheat bread	7 (12.1)	4.4 (0.8)	4.2	3.6	5.9
Wholegrain bread	8 (13.8)	7.5 (1.5)	7.7	5.3	9.6
Mixed breads	30 (51.7)	4.6 (0.8)	4.2	3.3	8.3
- *Mixed breads with seeds*	15 (25.9)	4.8 (1.5)	4.2	3.4	8.3
- *Mixed breads without seeds*	15 (25.9)	4.4 (0.6)	4.5	3.3	5.7

**Table 2:** The contribution of different bread types to the estimated habitual mean total bread (TB) intake in the Slovenian population.

Sex/Bread Category	Adolescents (*n* = 495)				Adults (*n* = 393)				Older Adults (*n* = 457)			
	24 HDR Non-Reporters *n* (%) *	Mean Intake g/day	Mean Contr. g/day	% of Daily Bread Intake (Rank)	24 HDR Non-Reporters *n* (%) *	Mean Intake g/day	Mean Contr. g/day	% of Daily Bread Intake (rank)	24 HDR Non-Reporters *n* (%) *	Mean Intake g/day	Mean Contr. g/day	% of Daily Bread Intake (Rank)
**Male**	252 (100)				183 (100)				223 (100)			
White wheat bread	94 (37.3)	127.5	80.0	54.6 (1)	104 (56.5)	144.3	62.6	39.4 (1)	144 (64.6)	120.2	42.6	24.9 (2)
Half-white wheat bread	218 (86.5)	98.7	13.3	9.2 (4)	148 (80.9)	134.8	25.9	16.3 (3)	174 (78.0)	128.1	28.1	16.4 (3)
Dark wheat bread	193 (76.6)	114.7	26.8	18.3 (2)	127 (69.4)	135.1	41.6	26.2 (2)	122 (54.7)	150.4	68.1	39.8 (1)
Mixed breads	231 (91.7)	129.2	10.8	7.3 (5)	173 (94.5)	139.0	7.6	4.8 (5)	205 (91.9)	117.2	9.5	5.5 (5)
Wholegrain	212 (84.1)	98.0	15.6	10.6 (3)	148 (80.9)	110.1	21.2	13.3 (4)	187 (83.9)	141.6	22.9	13.4 (4)
**Total**	6 (2.4) **		146.4	100	3 (1.6) **		158.9	100	4 (1.8) **		171.2	100
**Female**	243 (100)				210 (100)				234 (100)			
White wheat bread	116 (47.7)	97.2	47.7	52.1 (1)	134 (63.8)	99.5	36.2	34.6 (1)	158 (67.5)	102.5	33.4	25.4 (2)
Half-white wheat bread	210 (86.4)	83.8	11.4	12.4 (3)	183 (87.1)	93.8	12.1	11.5 (5)	186 (79.5)	114.1	23.5	18.0 (3)
Dark wheat bread	200 (82.3)	81.0	14.3	15.6 (2)	152 (72.4)	91.1	25.5	24.4 (2)	151 (64.5)	111.9	39.8	30.5 (1)
Mixed breads	214 (88.1)	91.9	11.0	12.0 (4)	183 (87.1)	93.9	12.1	11.5 (4)	209 (89.3)	103.1	11.1	8.5 (5)
Wholegrain	217 (89.3)	68.1	7.3	7.9 (5)	166 (79.0)	89.5	18.9	18.0 (3)	183 (78.2)	104.2	22.8	17.5 (4)
**Total**	8 (3.3) **		91.7	100	5 (2.4) **		104.8	100	10 (4.3) **		130.6	100

Note: * Participants who did not report intake of a certain type of bread in both 24 h recalls (HDR); ** *n* (%) True non-consumers of bread; Contr. = Contribution.

**Table 3:** The mean dietary fibre content in consumed bread and the determinants of dietary fibre intake identified using the Least Absolute Shrinkage and Selection Operator (LASSO).

Variable	Level	*n* (%)	Dietary Fibre Intake (g Fibre/100 g Bread Intake SD)	Selected Variables (LASSO) **
Age cohort	Adolescents	495 (36.8)	3.43 (0.86)	
	Adults	393 (29.2)	3.79 (1.32)	
	Older adults	457 (34.0)	3.63 (1.18)	
Sex	Male	658 (48.9)	3.50 (1.08)	X
	Female	687 (51.1)	3.72 (1.16)	
Region	Eastern	822 (61.1)	3.62 (1.10)	X
	Western	523 (38.9)	3.58 (1.18)	
Degree of urbanisation	Rural	752 (55.9)	3.56 (1.08)	X
	Intermediate	223 (16.6)	3.73 (1.17)	
	Urban	370 (27.5)	3.62 (1.20)	
Education *	Low	650 (76.5)	3.61 (1.10)	X
	High	200 (23.5)	4.06 (1.62)	
Employment *	Employed	236 (28.0)	3.86 (1.35)	X
	Unemployed	46 (5.5)	3.59 (1.37)	
	Student	34 (4.0)	3.80 (1.41)	
	Retired	528 (62.6)	3.63 (1.16)	
Household income	Low	335 (29.5)	3.53 (1.10)	
	Moderate	458 (40.3)	3.67 (1.14)	
	High	343 (30.2)	3.61 (1.15)	
IPAQ ***	Low	403 (30.3)	3.56 (1.11)	X
	Moderate	414 (31.2)	3.61 (1.17)	
	High	511 (38.5)	3.64 (1.12)	
Smoking *	Smokers	868 (69.6)	3.60 (1.11)	
	Non-smokers	380 (30.4)	3.62 (1.20)	

Note: * Variables only relevant for adults and older adults; ** Variables selected (X) based on the adaptive LASSO selection method; *** IPAQ = Levels of physical activity using the International Physical Activity Questionnaire scores.

**Table 4:** Mean habitual dietary fibre intake from different bread types and their contribution to total intake.

Sex/Bread Type	Adolescents (*n* = 495)		Adults (*n* = 393)		Older Adults (*n* = 457)	
	g/day	% of Daily Fibre Intake (Rank)	g/day	% of Daily Fibre Intake (Rank)	g/day	% of Daily Fibre Intake (Rank)
**Male**						
White wheat	2.17	46.0 (1)	1.70	34.3 (1)	1.17	24.2 (2)
Half-white wheat	0.50	10.5 (3)	0.97	19.6 (3)	1.05	21.8 (3)
Dark wheat	0.39	8.2 (5)	0.32	6.5 (5)	0.43	8.9 (4)
Mixed	0.48	10.2 (4)	0.34	6.9 (4)	0.42	8.7 (5)
Wholegrain	1.19	25.1 (2)	1.63	32.9 (2)	1.75	36.3 (1)
**Total dietary fibre**	4.73	100	4.96	100	4.82	100
**Female**						
White wheat	1.30	41.0 (1)	1.00	25.3 (2)	0.91	20.6 (2)
Half-white wheat	0.43	13.4 (4)	0.45	11.4 (5)	0.88	20.0 (3)
Dark wheat	0.40	12.5 (5)	0.53	13.4 (4)	0.37	8.4 (5)
Mixed	0.49	15.5 (3)	0.54	13.7 (3)	0.50	11.3 (4)
Wholegrain	0.56	17.6 (2)	1.43	36.2 (1)	1.75	39.7 (1)
**Total dietary fibre**	3.17	100	3.95	100	4.41	100

## Data Availability

The original contributions presented in this study are included in the Supplementary Materials. Additionally, bread composition data that support the findings of this study are available in DiRROS repository (URL: http://hdl.handle.net/20.500.12556/DiRROS-24055). This study applied SI.Menu 2017/18 national food consumption survey data, obtained as part of EFSA’s EU Menu project (Project URL: https://www.efsa.europa.eu/en/data-report/food-consumption-data; Structural Metadata: https://doi.org/10.5281/zenodo.1215992).

## Supplementary Materials

The following supporting information can be downloaded at: https://www.mdpi.com/article/10.3390/nu17223523/s1, Table S1: Descriptive statistics of dietary fibre content in breads sampled in small bakeries when requesting ”whole-grain bread”. Table S2: Descriptive statistics of dietary fibre content in sampled breads by bread type.
