# Supplementary material for: Bread Composition and Dietary Fibre Intake: Modelling Consumption Patterns and Substitution of White with Wholegrain Bread
Source: Nutrients. 2025 Nov 11;17(22):3523. doi: 10.3390/nu17223523 (PMC12655406; doi:10.3390/nu17223523)
Supplement: Supplementary file 1 [file nutrients-17-03523-s001.zip › nutrients-3896615-supplementary.pdf]

nutrients MDPI

## Supplementary materials

**Table S1.** Descriptive statistics of dietary fibre content in breads sampled in small bakeries when requesting ‘wholegrain’ bread.

| Classification | Bread categories | *n* (%) | Mean (SD) (g/100g) | Me-dian | Min | Max |
|---|---|---|---|---|---|---|
| All samples | Offered as “Wholegrain bread ” | 29 | 4.6 (1.2) | 4.2 | 3.2 | 8.9 |
| Samples after categorisation[1] | Whole-grain wheat/rye bread | 3 | 7.3 (1.5) | 7.2 | 5.9 | 8.9 |
| | Half-white wheat bread | 1 | 3.2 | | | |
| | Dark wheat breads | 2 | 4.1 (0.1) | 4.1 | 4.0 | 4.2 |
| | Mixed breads without seeds | 10 | 4.5 (0.7) | 4.6 | 3.4 | 5.7 |
| | Mixed breads with seeds | 13 | 4.3 (0.8) | 4.2 | 3.4 | 6.4 |

**Note:** [1]Categorisation of breads samples as explained in the methods section

**Table S2.** Descriptive statistics of dietary fibre content in sampled breads by bread type.

| Bread type | Purchase location | *n* (%) | Mean (SD) (g/100g) | Median | Min | Max |
|---|---|---|---|---|---|---|
| White wheat bread | All sources | 7 (12.1) | 2.9 (0.4) | 3.0 | 2.4 | 3.5 |
| | Large retail shops | 5 | 3.0 (0.4) | 3.0 | 2.5 | 3.5 |
| | Small bakeries | 2 | 2.7 (0.4) | 2.7 | 2.4 | 3.0 |
| Half-white wheat bread | All sources | 6 (10.3) | 3.7 (0.4) | 3.7 | 3.2 | 4.2 |
| | Large retail shops | 5 | 3.8 (0.4) | 3.8 | 3.3 | 4.2 |
| | Small bakeries | 1 | 3.2 | 3.2 | 3.2 | 3.2 |
| Dark wheat bread | All sources | 7 (12.1) | 4.4 (0.8) | 4.2 | 3.6 | 5.9 |
| | Large retail shops | 5 | 4.5 (0.9) | 4.7 | 3.6 | 5.9 |
| | Small bakeries | 2 | 4.1 (0.1) | 4.1 | 4.0 | 4.2 |
| Mixed breads | All sources | 30 (51.7) | 4.6 (0.8) | 4.2 | 3.3 | 8.3 |
| | Large retail shops | 7 | 5.2 (1.8) | 4.5 | 3.3 | 8.3 |
| | Small bakeries | 23 | 4.4 (0.8) | 4.2 | 3.4 | 6.4 |
| *Mixed breads with seeds* | All sources | 15 (25.9) | 4.8 (1.5) | 4.2 | 3.4 | 8.3 |
| | Large retail shops | 2 | 7.8 (0.7) | 7.8 | 7.3 | 8.3 |
| | Small bakeries | 13 | 4.3 (0.8) | 4.2 | 3.4 | 6.4 |
| *Mixed breads without seeds* | All sources | 15 (25.9) | 4.4 (0.6) | 4.5 | 3.3 | 5.7 |
| | Large retail shops | 5 | 4.2 (0.6) | 4.4 | 3.3 | 4.9 |
| | Small bakeries | 10 | 4.5 (0.7) | 4.6 | 3.4 | 5.7 |
| Wholegrain bread | All sources | 8 (13.8) | 7.5 (1.5) | 7.7 | 5.3 | 9.6 |
| | Large retail shops | 5 | 7.6 (1.7) | 8.1 | 5.3 | 9.6 |
| | Small bakeries | 3 | 7.3 (1.5) | 7.2 | 5.9 | 8.9 |
